# A bibliometric analysis of simulation-based learning in medical education: trends, gaps, and future directions

**DOI:** 10.3389/fmed.2025.1692991

**Published:** 2026-01-06

**Authors:** Afaf Sulaiman Alblooshi, Falah Mohammed AlMarzooqi, Taleb Mohammed Almansoori, Gamila Ahmed, Saif Al-Shamsi, Faten Abdullah AlRadini

**Affiliations:** 1 Department of Medical Education, College of Medicine and Health Sciences, United Arab Emirates University, Al Ain, Abu Dhabi, United Arab Emirates; 2 Department of Family Medicine, College of Medicine and Health Sciences, United Arab Emirates University, Al Ain, Abu Dhabi, United Arab Emirates; 3 Department of Radiology, College of Medicine and Health Sciences, United Arab Emirates University, Al Ain, Abu Dhabi, United Arab Emirates; 4 Public Services and Outreach Unit, National Medical Library, College of Medicine and Health Sciences, United Arab Emirates University, Al Ain, Abu Dhabi, United Arab Emirates; 5 Department of Internal Medicine, College of Medicine and Health Sciences, United Arab Emirates University, Al Ain, Abu Dhabi, United Arab Emirates; 6 Department of Family and Community Medicine, College of Medicine, Princess Nourah bint Abdulrahman University, Riyadh, Saudi Arabia

**Keywords:** bibliography, clinical competence, curriculum, educational technology, human, medical training

## Abstract

**Background:**

Simulation-based medical education (SBME) has become a cornerstone of modern healthcare training, enhancing clinical competence, decision-making, and patient safety. This study presents a bibliometric analysis of SBME literature from 2001 to early 2025, aiming to map publication trends, key contributors, thematic developments, and global research collaboration.

**Methods:**

A systematic search was conducted in Web of Science, Scopus, and PubMed. A total of 613 articles were screened, with 520 included for analysis. The Bibliometrix R package was used for trend analysis, author productivity, keyword co-occurrence, and collaboration network mapping.

**Results:**

SBME publications showed significant growth, peaking in 2024. Key contributors include Schijven M and Cook DA. Major research themes shifted from technical skill acquisition to non-technical competencies and technology-enhanced learning, including virtual reality and AI. Co-occurrence analysis revealed distinct thematic clusters and rising global collaborations, though disparities persist, particularly in underrepresented regions. A noted decline in 2025 output reflects partial-year data and is included only for context rather than trend interpretation.

**Conclusion:**

SBME research has evolved rapidly, driven by technological advances and global health priorities. Continued investment in equitable access, interdisciplinary collaboration, and outcome-based studies is vital to fully realize the transformative potential of simulation in medical education.

## Highlights

- **Rapid Growth and Thematic Shift:** SBME publications increased significantly from 2001 to 2024, evolving from technical skill training to include non-technical competencies, virtual reality, and AI-based innovations.
- **Global Inequities in Research Output:** Research is concentrated in high-income countries; low- and middle-income regions remain underrepresented, despite recent growth in Asia and the Middle East.
- **Influential Contributors Identified:** Authors like Schijven M and Cook DA lead the field, with most output concentrated in a few institutions and journals such as *Medical Teacher* and *BMC Medical Education*.
- **Technological Integration Accelerating:** Emerging trends emphasize AI-driven tutoring, VR/AR simulations, and machine learning analytics in SBME, reshaping how medical training is delivered and assessed.
- **Persistent Gaps and Future Needs:** Equity in access, real-world outcome validation, long-term impact studies, and localized adaptations remain underexplored and warrant prioritization.

## Introduction

Simulation-based learning (SBL) has become an integral component of modern medical education, offering a safe and controlled environment for healthcare professionals to acquire and refine clinical skills (1). This approach enhances clinical competence, decision-making abilities, and patient safety, providing significant advantages over traditional training methods (2, 3). By delivering immersive learning experiences, SBL enables trainees to practice high-risk procedures and manage complex clinical scenarios without exposing real patients to potential harm (4).

David M. Gaba, a pioneer in the field of medical simulation, emphasized that simulation is a technique, not merely a technology, designed to replicate substantial aspects of the real world in an interactive manner (5). His work laid the foundation for diverse applications of simulation in healthcare, from individual skills training to team-based crisis management and interprofessional education (5). The integration of simulation into medical curricula is crucial for preparing healthcare professionals to respond effectively in various clinical settings, thereby improving patient outcomes. However, despite its recognized benefits, gaps remain in understanding the long-term efficacy, optimal implementation strategies, and the role of emerging technologies in SBL (6).

Over the past decade, there has been a notable increase in research focusing on the clinical translational outcomes of simulation-based medical education (SBME). A bibliometric analysis of SBME literature from 2011 to 2021 reported a rise in publications from 48 in 2011 to 175 in 2021, with the United States leading in both the number of publications and citations (7). This increasing research trajectory highlights the expanding interest and investment in SBME as an essential strategy for advancing clinical training and optimizing patient outcomes. The growing body of literature underscores the recognition of simulation’s role in enhancing patient safety, minimizing medical errors, and reinforcing competency-based education (2).

Given the growing significance of SBL in medical education, a systematic understanding of its research landscape is essential. This study conducts a bibliometric analysis to identify current research trends, explore existing gaps, and propose future directions for optimizing SBL implementation. By analyzing the growth of research and key thematic areas, this study highlights underexplored aspects, such as the sustainability of simulation training and its integration across medical specialties. Despite these advancements, critical gaps remain in the existing literature, particularly in understanding the long-term retention of skills, cost-effectiveness, and the integration of emerging technologies such as virtual reality (VR) and artificial intelligence (AI) into simulation training. A bibliometric analysis can provide valuable insights into current research trends, highlight underexplored areas, and inform future directions for simulation-based education. This study aims to map the trajectory of SBME research, identify key themes, and propose recommendations to bridge existing gaps in the field.

## Materials and methods

### Study design and registration

This study employs a bibliometric analysis approach, using bibliometric indicators to quantitatively assess the research landscape of SBME. The analysis examines publication trends, leading contributors, productivity analysis, frequency analysis and co-occurrence analysis. The bibliometric analysis protocol was prospectively registered with the Open Science Framework (OSF) on 28th of February 2025.

### Data sources and search strategy

Data for this study were extracted from three major citation databases Web of Science (WoS) Core Collection, Scopus, and PubMed on the 27th of February 2025 by a medical librarian. The search strategy was developed using Medical Subject Headings (MeSH) and title/abstract fields to ensure comprehensive literature retrieval. Keywords related to SBME included:

1. “Simulation-based learning”
2. “Medical simulation”
3. “Educational technology”

Search strategies were customized for each database, with an English language filter applied. A detailed description of the search terms and strategy is provided in (Supplementary Material 1). Additionally, reference lists of included studies were manually screened for further eligible articles. Gray literature was excluded to maintain the quality and rigor of the analysis. The search was restricted to articles published between 2000 and 2025, ensuring coverage of recent advancements in SBME. Only peer-reviewed journal articles and conference proceedings were included, while book chapters, editorials, and non-peer-reviewed sources were excluded.

### Inclusion and exclusion criteria

*Inclusion criteria:*

1. Studies explicitly focusing on SBME in undergraduate, graduate, or continuing medical education.
2. Research analyzing the effectiveness, trends, or innovations in simulation-based training.
3. Publications indexed in Web of Science, Scopus, and PubMed with full citation records available.

*Exclusion criteria:*

1. Non-English publications.
2. Studies not related to simulation-based learning in medical education.
3. Editorials, commentaries, and non-peer-reviewed papers.
4. Studies on non-medical fields or unrelated simulation training.

### Data synthesis and analysis

Quantitative analysis was conducted using the Bibliometrix R package (version 4.3.1). The following variables were extracted and analyzed through the package: publication year (to track research trends over time), citation count (to assess impact), authors and affiliations (to identify leading researchers and institutions), journals (to determine where high-impact research is published), and keywords and thematic trends (to map the research focus over time).

To illustrate key research trends and knowledge gaps, the following analyses were conducted: co-authorship analysis (mapping collaboration networks among institutions, researchers and countries), keyword co-occurrence analysis (identifying emerging themes, evolving research topics, and research gaps), and citation analysis (highlighting the most influential publications and authors in SBME research). Journal-level indicators such as the local h-index (within corpus) were computed based on the analyzed dataset using the *Bibliometrix* R package. For network analyses, collaboration networks were constructed using country-level co-authorship data obtained through the *Bibliometrix* R package. Each node represented a country, and edges represented co-authorship links between countries. Network centrality measures, betweenness (the extent to which a node acts as a bridge between other nodes) and closeness (the inverse of the average distance to all other nodes) were calculated to identify influential countries and collaboration patterns within the SBME research network (8).

### Ethical considerations

This study involved secondary data analysis of publicly available literature, and no ethical approval was required. All data were retrieved from reputable databases, ensuring compliance with publication standards.

## Results

**FIGURE 1:**
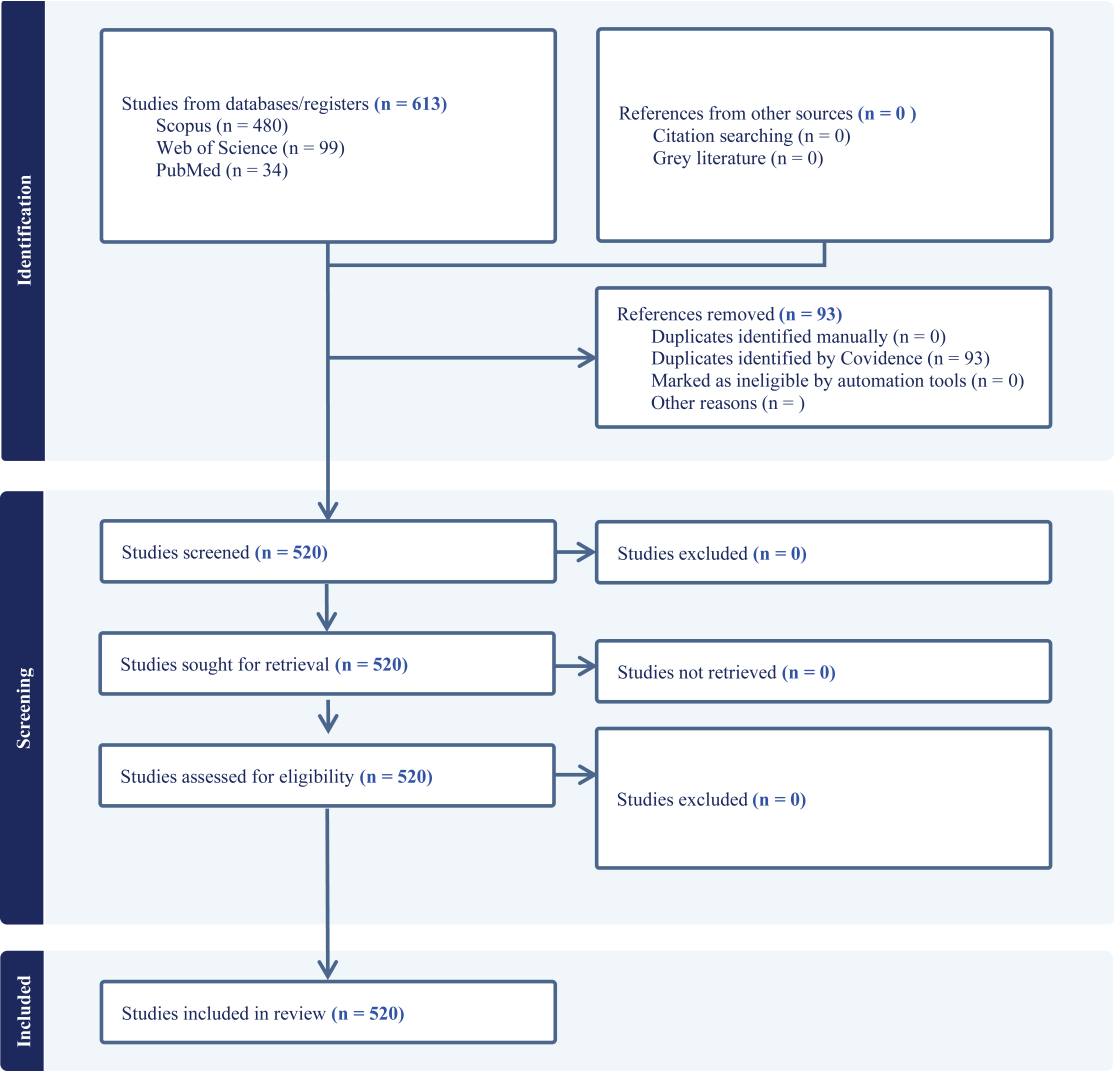
Database search strategy of simulation-based learning literature.

The initial search has identified a total of 613 studies across the three databases. After removing 93 duplicates (520 via Covidence), 520 studies were screened. In year 2000 there was no publication based of the search strategy (Figure 1).

### Publication trend

**FIGURE 2:**
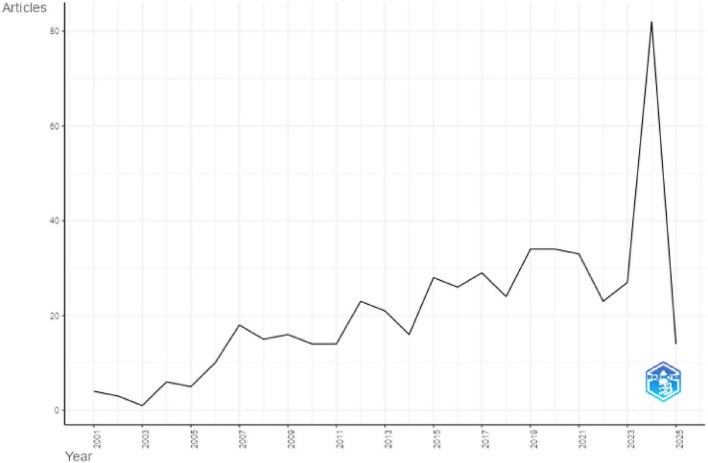
Annual scientific production from 2001 to 2025.

The analysis of annual scientific production from 2001 to 2024 reveals a general upward trajectory with notable fluctuations (Figure 2). The data in the figure indicates steady growth in scientific output from 2001 to 2019, peaking at 34 articles in both 2019 and 2020. After a slight decline in 2022 and 2023, there was an extraordinary surge in 2024, with 82 articles published, marking the highest output within the timeframe. The 2025 value reflects partial-year data collected in February and is included only for context, not for trend interpretation.

**FIGURE 3:**
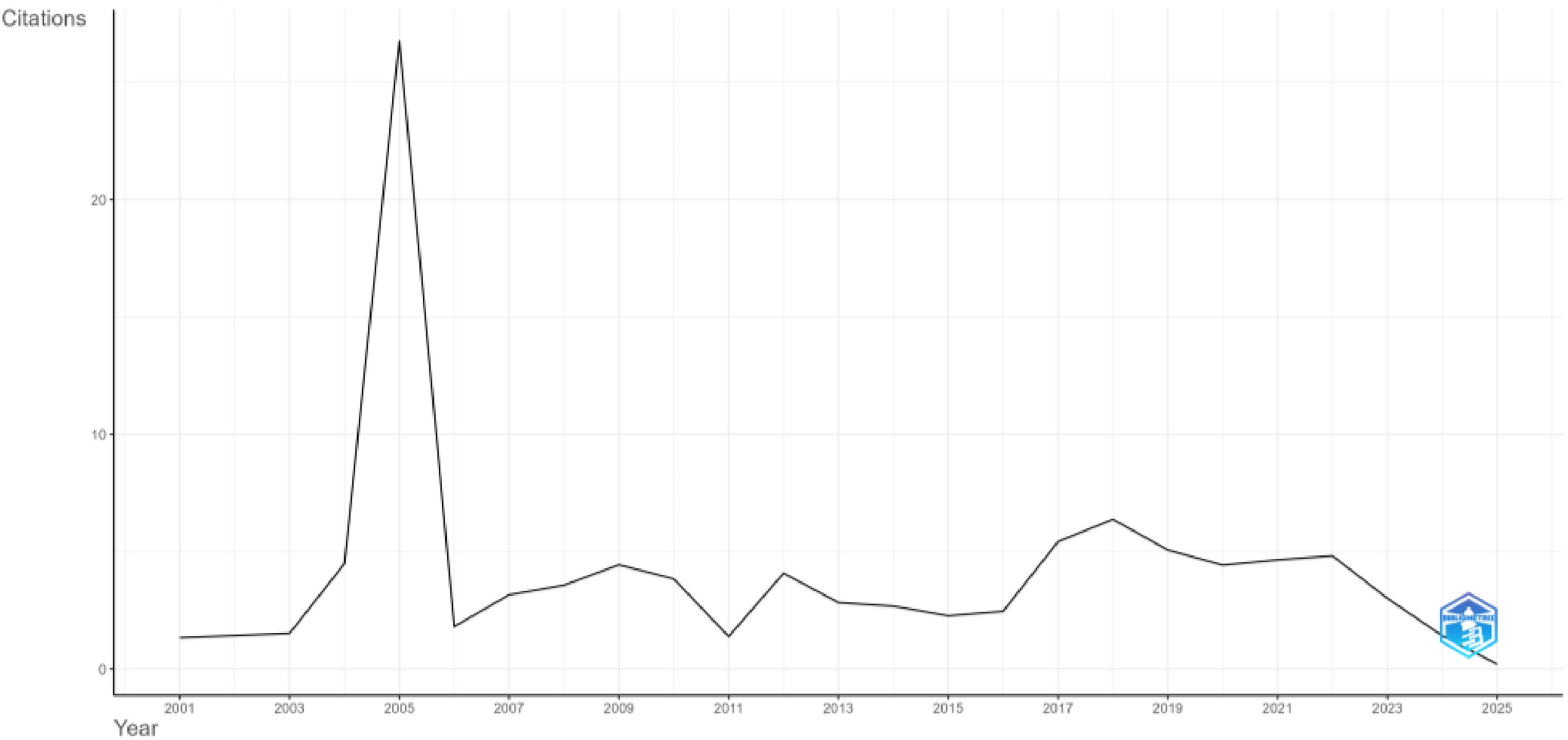
Average citation per year.

The average number of citations per article per year from 2001 to 2025 is 4.14 (9). The analysis highlights a significant peak in 2005, where the number of citations per article per year reached 26.76, a value influenced by the very small number of publications that year (Figure 3). The average number of total citations per article from 2001 to 2025 is 58.97, reflecting substantial citation activity across the corpus. The highest citation impact was observed in 2005, where the number of citations per article reached 562, an artifact of one or two highly cited papers within a small cohort. Figure 3 represents the average citations per year, highlighting the citation spike in 2005 and the subsequent decline, while the sharp drop in 2025 reflects data collection occurring in February rather than a full year’s accumulation.

**FIGURE 4:**
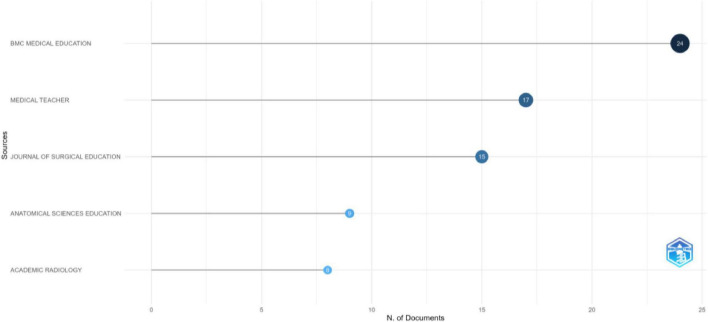
Most relevant sources.

The analysis of journal publications shows that the majority of research has been published in leading medical education and surgery-related journals, with “*BMC Medical Education*” (24 articles) and “*Medical Teacher*” (17 articles) being the most common publication venues (Figure 4). Within our dataset, *Medical Teacher* holds the highest local h-index (within corpus) of 12, indicating strong influence among the SBME studies analyzed. *BMC Medical Education* and *Journal of Surgical Education* follow closely with local h-indices of 11 and 10, respectively, reflecting their relevance in surgical and general medical education. *Anatomical Sciences Education* has a lower local h-index of 7 but shows a strong total citation count of 1,046, suggesting a highly cited but specialized niche. Other notable journals include *Academic Emergency Medicine* and *Academic Radiology*, both with local h-indices of 6, highlighting their contribution to specialized areas in medical training. The presence of newer journals like *Cureus Journal of Medical Science* (local h-index: 4, Publication Year start: 2020) suggests an emerging role in medical publishing (Supplementary Material 2).

**FIGURE 5:**
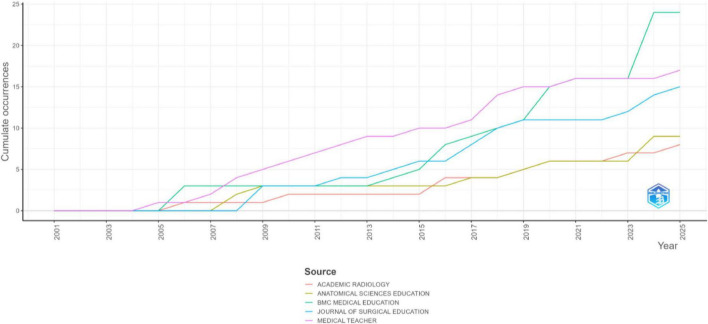
Sources’ production over time.

The growth of simulation-based medical education research across five major journals: *BMC Medical Education, Medical Teacher, Journal of Surgical Education, Anatomical Sciences Education*, and *Academic Radiology*; has expanded significantly from 2001 to 2025 (Figure 5). No publications on simulation-based education appeared in these journals until 2005, when *Medical Teacher* published its first article on the subject. Research output gradually increased from 2006 onward, with a notable rise in *BMC Medical Education* and *Medical Teacher*, reflecting a growing academic interest in medical simulation. The cumulative occurrence graph (Figure 5) further highlights this trend, showing a sharp increase in publications after 2015 across all journals. *BMC Medical Education* and *Medical Teacher* exhibited the steepest inclines, establishing themselves as primary outlets for simulation-based medical education research. The *Journal of Surgical Education* also experienced significant growth, particularly after 2009, underscoring the increasing adoption of simulation in surgical training. Between 2016 and 2025, the field witnessed its most rapid expansion, with *BMC Medical Education* reaching 24 publications by early 2025. *Medical Teacher* and the *Journal of Surgical Education* followed similar trajectories, while *Academic Radiology* and *Anatomical Sciences Education* showed more gradual yet consistent growth.

### Leading contributor and productivity analysis

The most influential authors in medical simulation research, based on publication count, are Schijven M (10–16), Cook DA (6, 17–21), and Aggarwal R (22–26) (Supplementary Material 3), with Schijven M leading at seven publications, followed by Cook DA with six and Aggarwal R with five. Several other researchers, including Del Maestro, R (27–30), Konge L (31–34), Ledwos N (27–30), Mirchi N (27–30), Winkler-Schwartz A (27–30), Yilmaz R (27–30), and Bissonnette V (28–30), have also contributed notably, with each author having four publications, except for Bissonnette V, who has three. This distribution highlights a core group of scholars who have significantly shaped the field, particularly through research on virtual reality, simulation-based training, and educational technologies in medical education. *Cook DA* has played a crucial role in simulation-based medical education, adaptive learning, and virtual patient simulation, as highlighted by his 2025 work on creating virtual patients using large language models (35). *Aggarwal R* has been instrumental in integrating simulation in surgical oncology and patient safety, particularly with a highly cited 2010 paper on training and simulation for patient safety (24).

The publication list further reinforces the importance of AI-driven simulation in medical education. Authors like *Mirchi N, Yilmaz R*, and *Winkler-Schwartz A* have contributed significantly to machine learning and Artificial Intelligence (AI)-based medical simulations, particularly with their 2021 research on AI tutoring versus expert instruction for surgical training (36). The *Journal of Surgical Education, JAMA Network Open*, and *PLOS One* have emerged as major platforms for these advancements, showcasing the field’s rapid evolution toward AI-assisted and virtual reality (VR) simulation training.

The ranking of the institutional contributions to medical and health sciences research by publication count (Supplementary Materials 4, 5). The University of Washington leads with 31 publications, followed by McGill University and the University of Toronto with 30 each, Harvard Medical School with 24, and the University of Calgary with 23. Major contributors include the Mayo Clinic, Stanford University, and Imperial College London. The visualization provides an empirical basis for analyzing academic publishing trends in healthcare.

**FIGURE 6:**
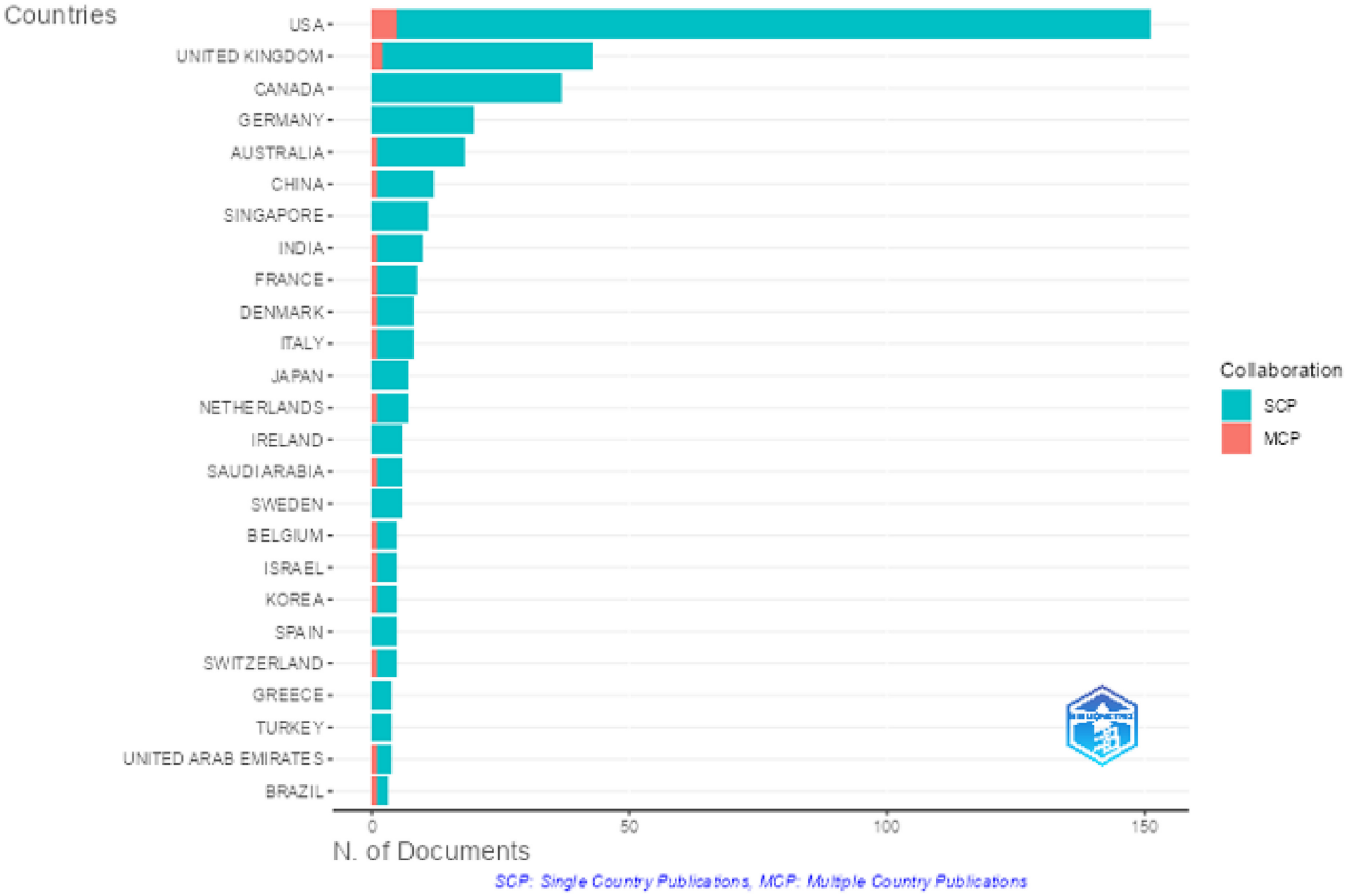
Corresponding author’s countries.

Geographically, corresponding authors are predominantly from the USA, followed by the UK and Canada, which collectively contribute a significant portion of publications (Figure 6). While single-country publications (SCP) dominate, multiple-country collaborations (MCP) remain relatively low, particularly in developing regions (24).

### Global and institutional contributions to SBME

**FIGURE 7:**
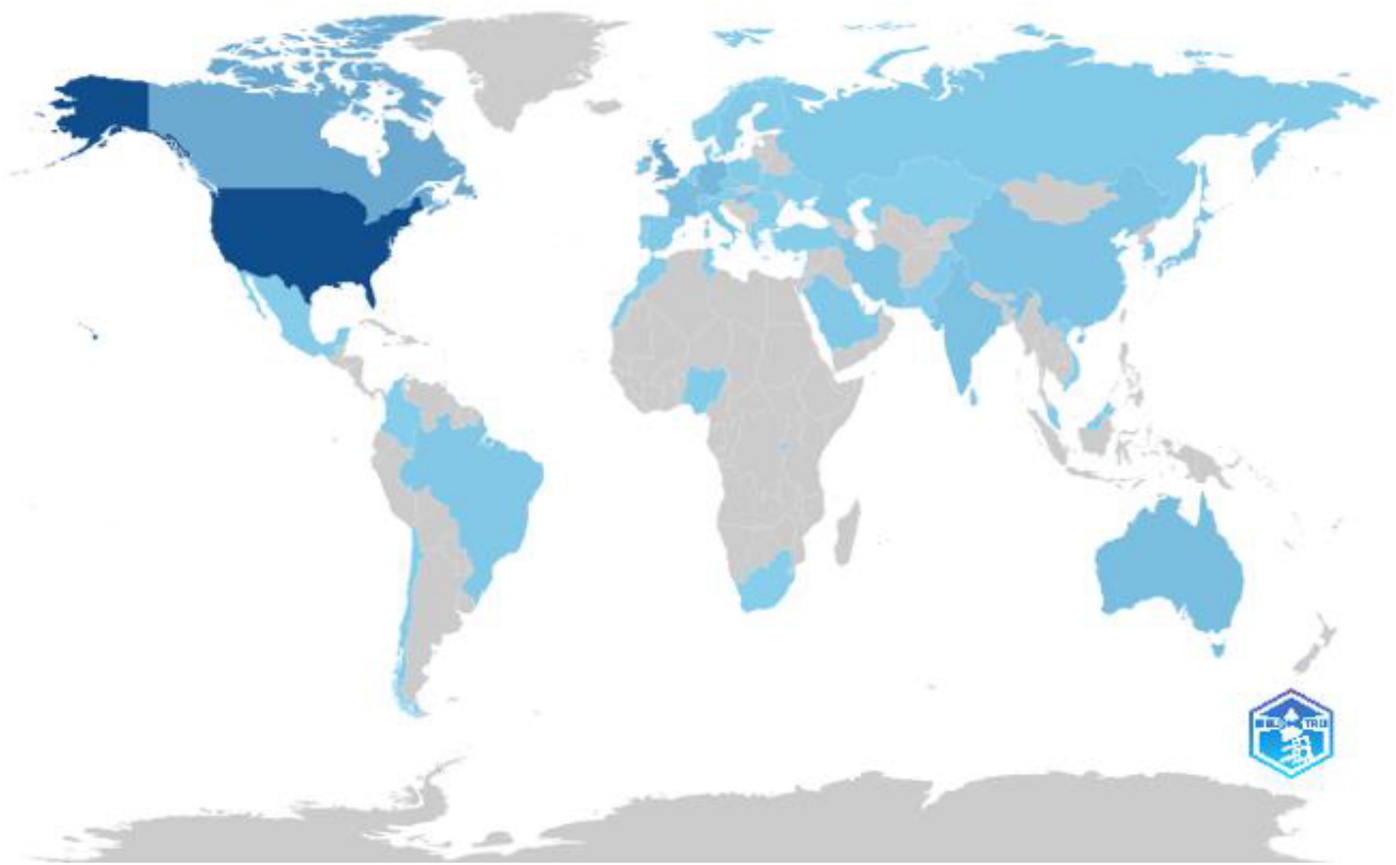
Country scientific production.

The global distribution of scientific production (Figure 7) shows a strong concentration of research output in high-income countries, with the United States leading at 296 publications. The United Kingdom and Canada follow with 90 and 79 publications, respectively, supported by robust academic infrastructures. European nations such as Germany with 46, France with 35, and Australia with 34 maintain a steady presence, reflecting their commitment to scientific advancement. Emerging economies like India with 27, China with 23, and Iran with 21 demonstrate increasing contributions, indicative of growing investments in higher education and innovation. The Middle East is represented by Saudi Arabia with 9 and the United Arab Emirates with 6, highlighting a rising research capacity. African nations such as Nigeria with 6 and Rwanda with 2 features in the dataset but remain underrepresented, reflecting ongoing challenges in research funding and infrastructure.

### Leading global citations in SBME

Simulation-based education has produced highly influential research, with certain publications achieving significant academic impact (Supplementary Material 6). Citation analysis identifies both foundational studies and emerging trends. The most cited paper is Issenberg S B (2005, MED TEACH) (2) with 2,514 citations, showing its key contribution to medical education. More recent works, such as Jensen L (2018, EDUC INF TECHNOL) (37) with 683 citations and Moro C (2017, ANAT SCI EDUC) (38) with 617 citations, show high annual citation rates. Normalized citation impact underscores their influence, with Jensen L (2018) (37) at 13.38 and Moro C (2017) (38) at 12.61.

### Frequency analysis of SBME keywords

**FIGURE 8:**
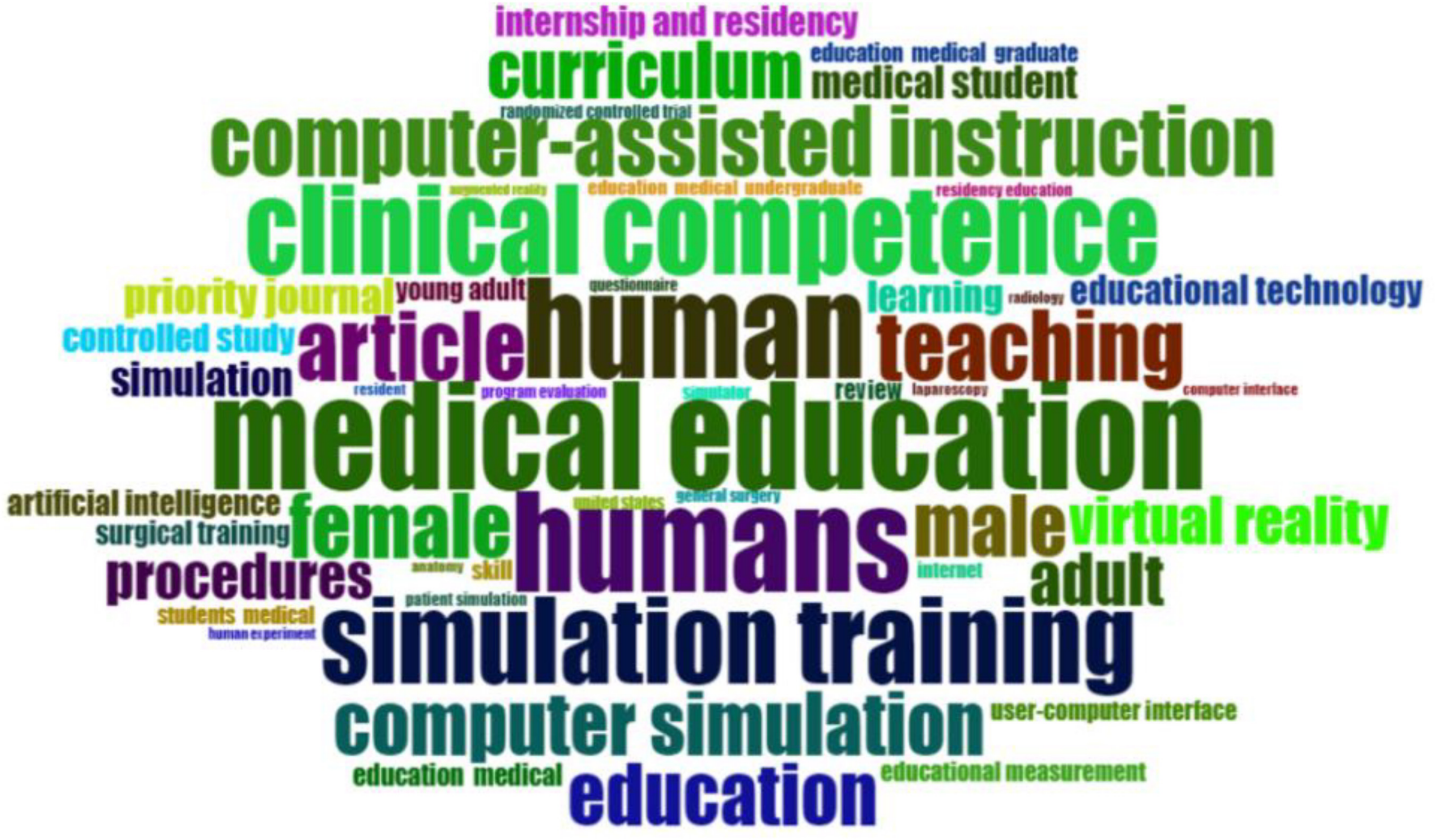
Word cloud of the key terms in medical education research.

The frequency analysis of key terms in medical education research (Figure 8) highlights the most frequently occurring terms in simulation-based medical education (SBME) literature. Dominant terms include medical education, humans, clinical competence and simulation training, underscoring the central themes of human-centered, competency-based training. Keywords such as computer-assisted instruction, computer simulation, and virtual reality reflect the integration of technology into medical curricula.

The prominence of female and male indicates a consistent focus on gender representation in research populations. Additionally, terms like teaching, curriculum and education reinforce the structured and pedagogical nature of the field. The appearance of procedures, task performance and laparoscopy suggests an emphasis on hands-on, skills-based learning methodologies.

Emerging trends are also evident, with keywords such as artificial intelligence and machine learning pointing toward the increasing role of AI in medical education. Overall, the word cloud illustrates both foundational elements and evolving innovations shaping the SBME research landscape.

### Co-occurrence analysis

Co-occurrence analysis (Supplementary Material 7) reveals two major research clusters: a *red cluster* focused on *core medical education topics* (e.g., *clinical competence, curriculum*, and *teaching*) and a *blue cluster* centered on *simulation and digital education technologies*. Timeline analysis shows sustained relevance of *traditional medical education topics*, alongside a rapid increase in *AI, machine learning*, and *virtual reality* research.

**FIGURE 9:**
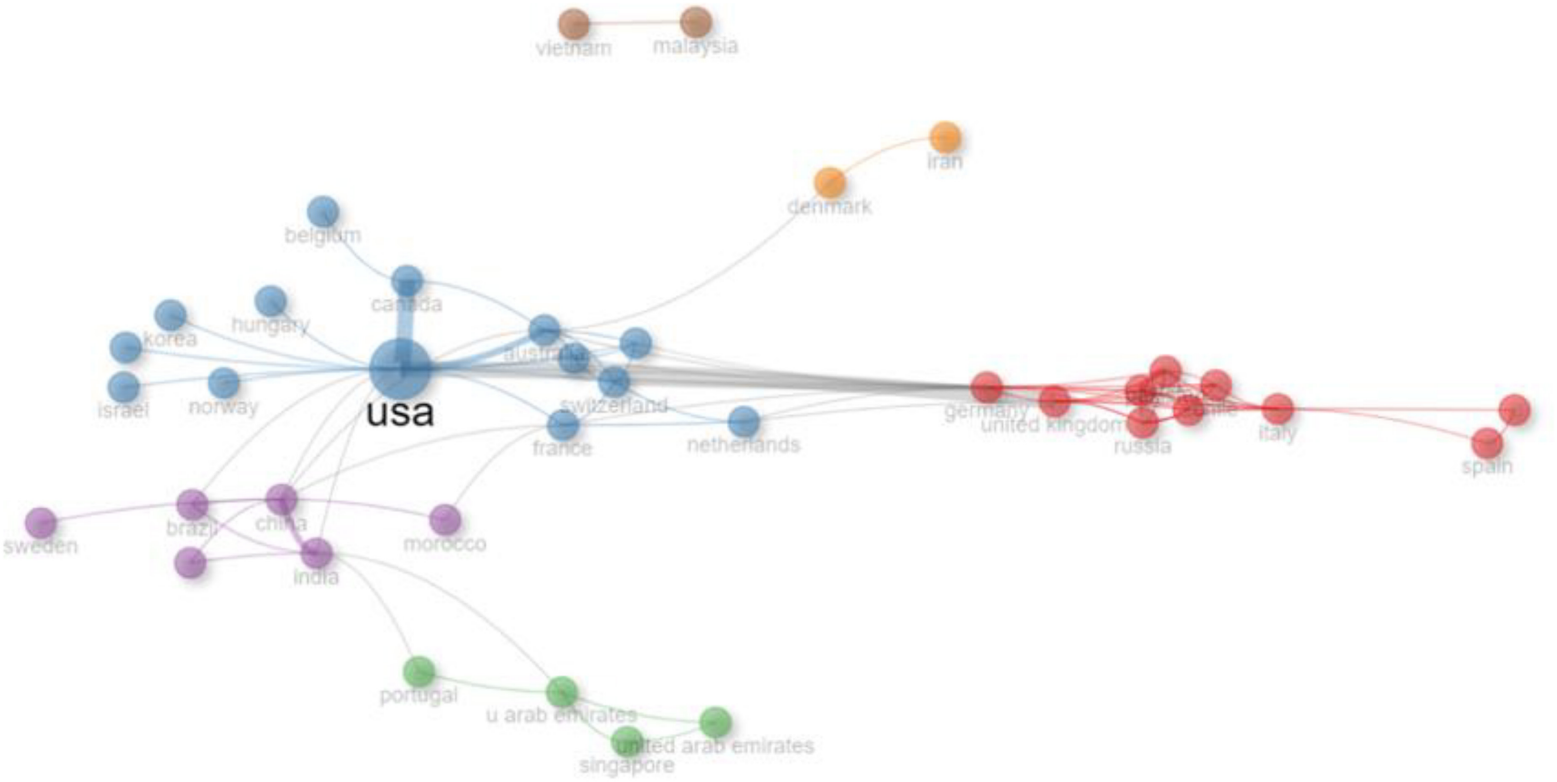
Co-occurrence network of collaboration in medical simulation research.

The co-occurrence network analysis (Figure 9) identifies multiple clusters of research collaboration among different countries. The largest hub in the network is the United States, with the highest betweenness centrality of 333.59, indicating its crucial role in connecting various countries in research collaborations. Cluster one includes European and South American countries such as the United Kingdom, Germany, Italy, and Spain, with Germany and the UK playing significant intermediary roles. Cluster two consists of North American, Australian, and select European countries, with Australia having a high betweenness score of 79.37, signifying its importance in linking different research nodes. Cluster three primarily comprises Middle Eastern and Asian nations, including the UAE and Singapore, whereas cluster four encompasses India, China, and Brazil, with India and China having substantial betweenness values. Notably, Malaysia and Vietnam in cluster six exhibit exceptionally high closeness scores of one, suggesting they have direct access to many other nodes, though they lack strong intermediary roles.

## Discussion

### Evolution of publication trends in SBME

Over the past quarter-century (2001–2025), simulation-based medical education (SBME) has evolved from a niche pedagogical innovation to a cornerstone for competency-based training and patient safety. This bibliometric analysis reveals not only a steady rise in publications but also a shift in research priorities; from validating simulation as an educational method to optimizing its integration, scalability, and impact on health outcomes. Such evolution mirrors broader changes in health professions education, where experiential learning and patient safety frameworks have become central to curricular reform (2, 3).

### Authorship, productivity and scholarly influence

The author and institutional patterns suggest a “core-periphery” structure typical of a mature research field. A relatively small group of prolific authors and well-resourced centers has produced a disproportionate share of the literature, shaping research agendas around surgical simulation, virtual reality, and technology-enhanced training (10–23). These core groups have often worked in stable collaborations, enabling longitudinal programs of research, curriculum development, and validation studies. At the same time, the increasing number of unique authors over time indicates widening participation, with clinicians, engineers, computer scientists, and educational researchers contributing to SBME from different disciplinary perspectives. Going forward, nurturing emerging scholars and broadening mentorship networks will be important for diversifying the questions asked and the methods used (36)

### Influential studies and citation dynamics

The citation landscape is dominated by a small number of highly cited systematic reviews, conceptual papers, and technology-focused trials that have set the direction of SBME for many years (2, 3, 5, 6, 9–11, 17–19, 27–30, 38–40). These “anchor” publications have shaped how educators think about fidelity, deliberate practice, curriculum design, and outcome measurement. The prominence of review articles and guidelines also indicates the field’s early need for synthesis and consensus. More recent citation patterns show growing interest in immersive technologies, AI-enabled assessment, and translational outcomes, signaling a shift from establishing efficacy to understanding mechanisms, cost-effectiveness, and impact on clinical practice (7, 27–30, 35, 38, 41). A key next step will be to strengthen links between simulation exposure and patient-level or system-level outcomes, beyond self-reported confidence and short-term performance (3, 4, 39).

### Shifting themes and emerging technologies in SBME

Keyword and thematic analyses show that SBME has expanded far beyond basic skills training to encompass complex domains such as interprofessional teamwork, communication, crisis resource management, and systems thinking (1, 5, 10, 11, 24, 39, 40). In parallel, emerging terms such as artificial intelligence, machine learning, virtual reality, and augmented reality signal a transition toward more immersive and data-rich learning environments (14, 15, 27–30, 35, 38). AI-driven simulators and virtual patients now offer adaptive scenarios, automated feedback, and scalable training opportunities, potentially lowering barriers to access over time (27–30, 35). However, these advances also raise questions about cost, infrastructure, faculty readiness, and regulatory oversight, particularly in settings where basic simulation facilities are still being established. Comparative effectiveness studies that examine when high-tech solutions add value over lower-tech or hybrid models will be crucial for rational and equitable adoption (3, 6, 17–19).

### Thematic and collaboration networks in SBME

Co-occurrence and collaboration networks reveal two broad types of “connection” shaping the field: thematic links between concepts and structural links between countries and institutions. Thematically, SBME has moved from a focus on technical skill acquisition to a broader emphasis on patient safety, non-technical skills, and ultimately the translation of simulated performance into real-world outcomes (22, 39, 40). Structurally, the network is anchored by a few high-income countries that act as hubs for international collaboration (7, 42–44). While these hubs help to disseminate innovations and standards, the relatively low proportion of multiple-country publications suggests that collaboration is not yet fully leveraged as a mechanism for capacity building and agenda setting in under-resourced regions (42, 45, 46). Intentional partnerships that position LMIC institutions as equal collaborators—rather than peripheral data sites—could broaden perspectives and ensure that SBME research addresses diverse educational, cultural, and health-system priorities (45, 46).

### Implications for practice and future directions

Taken together, these patterns position SBME as a mature yet rapidly evolving field. The main challenge is no longer to prove that simulation “works,” but to determine how to implement it efficiently, equitably, and sustainably across varied contexts. Future research and practice should:

1. *Strengthen outcome linkage:* Design studies that connect simulation exposure to clinical behavior, patient outcomes, and system performance, using robust and longitudinal designs (3, 4, 39).
2. *Advance equity and access:* Develop and evaluate models that are feasible in resource-constrained settings (e.g., low-cost simulators, shared regional centers, blended online-in-person formats), and explicitly examine equity impacts of new technologies (42, 45, 46).
3. *Integrate and evaluate emerging technologies*: Move from proof-of-concept to implementation science for AI, VR, and AR in SBME, including faculty development, ethical use of learner data, and cost-effectiveness analyses (14, 15, 27–30, 35, 38).
4. *Broaden methodological approaches:* Combine bibliometric mapping with qualitative, mixed-methods, and participatory designs to capture how culture, policy, and organizational factors influence SBME adoption and impact (8, 9, 36, 43, 44).

By focusing on these directions, SBME can continue to evolve from a collection of successful local innovations into a globally relevant, evidence-informed approach that meaningfully improves healthcare education and, ultimately, patient care.

### Limitation

One notable limitation is the partial-year nature of the 2025 data. The 2025 values are included only for transparency, as they represent early-year data rather than a full publication cycle and are not used to interpret trends. The main trend interpretation is therefore based on data up to 2024.

## Conclusion

This bibliometric analysis illustrates a vibrant and evolving SBME landscape from 2001 to 2025. The field has seen exponential growth in scholarly output, driven by key milestones such as the mid-2000s validation of simulation pedagogy and the early 2020s push for innovation under pandemic pressures. Influential champions of SBME individuals, institutions, and countries have established a robust foundation that is now broadening via global collaboration and interdisciplinary synergy. Thematically, SBME research has progressively shifted from demonstrating “does it work?” to exploring “how to make it work best,” embracing new technologies and educational paradigms along the way. As SBME enters the age of AI and immersive virtual training, its core mission endures: to enhance the competency, safety, and readiness of healthcare professionals. The continued monitoring of publication trends, impact metrics, and thematic directions will be crucial in guiding educators and policymakers. Such insights ensure that the collective knowledge is leveraged to address remaining gaps from ensuring equitable access to simulation training, to rigorously linking simulation learning to patient outcomes thereby fully realizing the promise of simulation-based medical education in improving healthcare delivery (10, 19).

## Data Availability

The original contributions presented in the study are included in the article/Supplementary Material, further inquiries can be directed to the corresponding author.
